# Optimal Identification of Muscle Synergies From Typical Sit-to-Stand Clinical Tests

**DOI:** 10.1109/OJEMB.2023.3263123

**Published:** 2023-03-29

**Authors:** Simone Ranaldi, Leonardo Gizzi, Giacomo Severini, Cristiano De Marchis

**Affiliations:** Deparment of Industrial, Electronics and Mechanical EngineeringRoma Tre University 19012 00154 Rome Italy; Institute for Modelling and Simulation of Biomechanical SystemsUniversity of Stuttgart 9149 70174 Stuttgart Germany; School of Electrical and Electronic EngineeringUniversity College Dublin 8797 4 Dublin Ireland; Department of EngineeringUniversity of Messina 18980 98122 Messina Italy

**Keywords:** Sit-to-stand, muscle synergies, surface electromyography, biomedical signal processing, clinical test

## Abstract

*Goal:* The goal of this manuscript is to investigate the optimal methods for extracting muscle synergies from a sit-to-stand test; in particular, the performance in identifying the modular structures from signals of different length is characterized. *Methods:* Surface electromyography signals have been recorded from instrumented sit-to-stand trials. Muscle synergies have then been extracted from signals of different duration (i.e. 5 times sit to stand and 30 seconds sit to stand) from different portions of a complete sit-to-stand-to-sit cycle. Performance have then been characterized using cross-validation procedures. Moreover, an optimal method based on a modified Akaike Information Criterion measure is applied on the signal for selecting the correct number of synergies from each trial. *Results:* Results show that it is possible to identify correctly muscle synergies from relatively short signals in a sit-to-stand experiment. Moreover, the information about motor control structures is identified with a higher consistency when only the sit-to-stand phase of the complete cycle is considered. *Conclusions:* Defining a set of optimal methods for the extraction of muscle synergies from a clnical test such as the sit-to-stand is of key relevance to ensure the applicability of any synergy-related analysis in the clinical practice, without requiring knowledge of the technical signal processing methods and the underlying features of the signal.

## I. Introduction

Sit-to-stand test is a well estabilished clinical test for the staging and the detection of different pathologies mostly linking the speed and the regularity of repeated movements to the independence of the patient during activities of daily living [1]. These tests are typically carried out by clinicians recording semi-quantitative informations about the movement such as the time that is needed for executing 5 times the movement (*5 times sit to stand*) or the number of complete cycles that can be realized in 30 seconds (*30 seconds sit to stand*); recently, some instrumented versions of the sit-to-stand test have been introduced [2], [3], [4], in order to increase the amount of information that can be extracted and processed in the clinical scenario.

In order to ensure the clinical validity of the results coming from the instrumented tests, while avoiding additional burden by the clinicians to perform the test, these implementations are typically realized using simple wearable sensors [2] or conventional cameras [4]. These instrumental protocols are typically coupled with algorithms that are specifically designed to work in a limited time frame such as the ones defined by the aforementioned non-instrumented tests. Any of the mentioned simple implemetations typically rely on quantitative information to identify with better performance the clinical indicators that are evaluated by the clinicians, as well as adding additional insights on the kinematic strategies of movement control during the task. Other approaches use a more complex setup to measure more detailed kinematic variables [3] or muscle activity [5], [6] using instrumentation that is typical of a movement analysis research laboratory.

Among the analyses that have been carried out for the muscle activity, the investigation of how muscle coordinate to achieve the sit-to-stand movement has become prominent in the literature in the last years [7], [8], [9], [10], [11], [12]; the results presented in these studies exploit the synchronous muscle synergy model [13] to identify coordinated patterns of muscle activity that are related to specific biomechanical functions or features of the movement. The information coming from muscle synergy analysis can be exploited for integrating the kinematic and kinetic characterization of movement [8], [12], to identify the level of motor impairment in pathologies [7], [10], [11], to track progress in therapies [9] or to drive muscoloskeletal simulations [14]. In all these applications, the correct identification of the synergistic structure is of key importance and the achievement of such an accuracy typically relies on a number of replications of the movement that exceeds the length of the standard tests that are adopted in the clinical environment [15], [16], [17].

The accurate and thorough extraction of muscle synergies from multi-muscle surface electromyography (sEMG) recordings requires a precise tuning of the methodologies that process and factorize the raw signals. The importance of a correct methodological workflow has already been studied for the sEMG amplitude estimation [18], [19], [20], for factorization algorithm performance [21] or for model selection criteria [22], [23]. In all the mentioned study, the main result is that of a critical importance of having either objective or optimal methods for the synergy analysis in order to capture clinically relevant information from the coordinated structure of muscle activity; non optimality of the methods adopted have been proven to yield errors in the identification of both the number and the structure of the muscle synergies, thus generating wrong interpretation of their clinical meaning. In this scenario, in which clinicians are not necessarily required to have advanced technical knowledge related to the extraction algorithms, an optimal method is characterized by its capability of generating robust results that are not dependent on any setting (i.e. free parameters) that must be defined prior to the analysis.

The optimality of the processing methods does not by itself ensure a good estimation of the synergies underlying movement control; in addition to the processing choices, the selection of the length of the signals (in terms of number of repetition of the movement) has a significant effect on the outcomes [15], [16]. While in general terms having longer signals should improve the quality of any analysis, this kind of conditions might affect the way in which the movement is performed, after minutes of repetitive movements, as well as introducing non-stationarities due to accomodation and fatigue. This suggest that, in order to have a reliable and ecological assessment of muscle synergies in standard clinical tests, it is important to understand how using relatively short signals (i.e. shorter than the ones used in typical synergy-related experiments) affects the results.

In this paper a comparison between the accuracy in extracting muscle synergies from a relatively long (i.e. 30 seconds) and one relatively short (i.e. 5 times) sit-to-stand clinical trial is presented, aiming to provide a minimal length of a standard test that can identify correctly muscle synergies while remaining ecological in nature. Moreover, the signals are also differentiated in sit-to-stand only, stand-to-sit only and cyclical (both phases), in order to check whether the information about coordinated muscle structures can be completely determined by the control strategies of one of the two phases.

## II. Materials and Methods

### A. Experimental Protocol

**Fig. 1.**
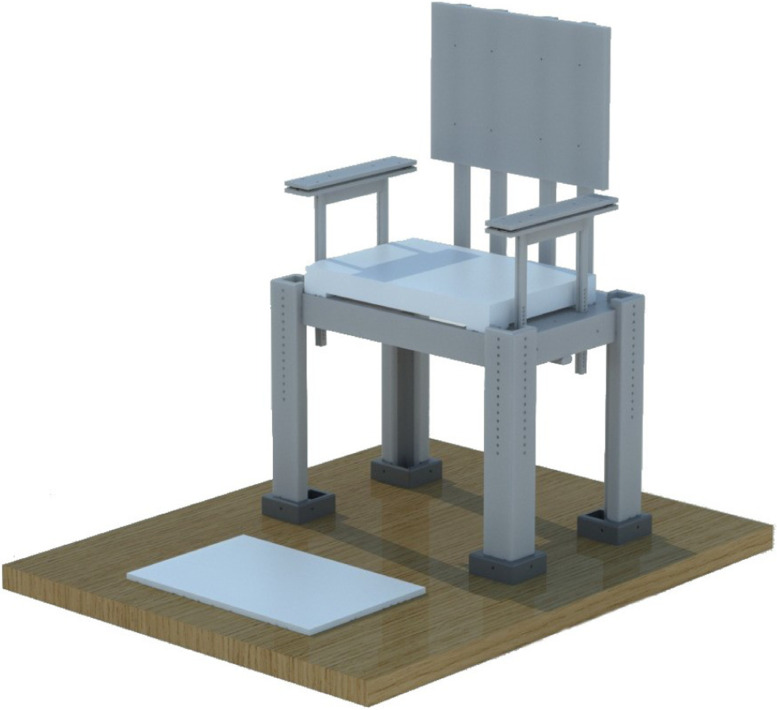
The instrumented chair used for the experiments.

The experiment has been carried out using the instrumented chair in Fig. 1. The subject executed a *30 seconds sit-to-stand* test in a standardized posture with the arms folded across the chest. In order to simulate the *5 times sit-to-stand* test, we extracted 5 cycles from the longer trial.

The instrumented chair is equipped with two force plates (BTS P6000, BTS Bioengineering, Milan, Italy), embedded in the design of the chair itself, representing the seating and standing planes. Kinematic data were recorded with a BTS SMART DX6000 system (BTS Bioengineering, Milan, Italy) and surface electromyography has been recorded at a sampling frequency of 1 kHz with a BTS FREEEMG System (BTS Bioengineering, Milan, Italy) from the following muscles of the right leg: *Soleus* (SOL), *Gastrocnemius Medialis* (GMED), *Peroneus Longus* (PER), *Tibialis Anterior* (TA), *Vastus Lateralis* (VL), *Rectus Femoris* (RF), *Biceps Femoris* (BF) and *Gluteus Maximus* (GLU).

In total, 13 healthy subjects (11 M and 2 F, $36.9 \pm 10$ years, $177.2 \pm 6.6$ cm, $79 \pm 11.7$ kg) participated to the experiment. All the trials were performed at the laboratory of bioengineering Biolab3 of Roma Tre University. The whole experimental procedure was conducted according to the principles of the Declaration of Helsinki, and it was approved by the ethics committee of the Roma Tre University.

### B. Event Identification and Tested Conditions

A complete cycle has been defined from the start of the sit-to-stand movement to the start of the following one. The movement is then composed of two phases:

• *Upward*, from sit to stand, the first phase of the cycle.
• *Downward*, from stand to sit, the concluding phase of each cycle.

The phases have been identified from the speed vector of the *sacrum* marker; The instant of change in antero-posterior speed direction is considered to be the phase transition event.

The muscle synergy analysis has been then conducted in six different conditions

• *30 s*, using the complete *30 seconds sit-to-stand* trial
• *30upward*, using all the upward phases for the *30 seconds sit-to-stand*
• *30downward*, using all the downward phases for the *30 seconds sit-to-stand*
• *5tsts*, using the complete *5 times sit-to-stand* trial
• *5upward*, using all the upward phases for the *5 times sit-to-stand*
• *5downward*, using all the downward phases for the *5 times sit-to-stand*

### C. Synergy Extraction

For synergy extraction, sEMG signals have been filtered for denoising with a bandpass filter (40–350 Hz, $4\text{th}$ order Butterworth). The amplitude envelope of the signal has been extracted via rectification and low-pass filtering (10 Hz, $4\text{th}$ order Butterworth). Each envelope has then been normalized in amplitude to its median peak value across all cycles. All the analysed cycles were organized in an $N_{s}$-by-$N_{m}$ matrix (where $N_{m}$ is the number of muscles, and $N_{s}$ the number of samples), in which each column is the concatenation of the envelope of a muscle coming from the different cycles.

Synergies have been extracted via Non-Negative Matrix Factorization [24] and the number of synergies $N_{syn}$ has been selected from each signal according to the optimal Akaike Information Criterion (AIC) method presented in [22]. The modified AIC criterion is written in the mathematical form as:

\begin{align*}
AIC(k) \!=&\!\! \sum _{i=1}^{N_{m}}\sum _{j=1}^{N_{s}} \frac{\left(M_{i,j} \!-\! \hat{M}_{i,j}\right)^{2}}{\sigma ^{2}_{M} \!+\! \sigma ^{2}_{SDN;i,j}} \!+\! 2kN_{m}\\
&\!+\!2\sum _{i=1}^{k}\sum _{j=1}^{L_{e}}N_{DoF;i,j} \tag{1}
\end{align*}

where $M$ and $\hat{M}$ are the original and reconstructed data, respectively, $k$ is the number of synergies and $N_{DoF}$ is the number of degrees of freedom of the model. In this equation, the noise in the original data is approximated to be corrected by an additive signal dependent term $\sigma ^{2}_{SDN;i,j}$, and the actual number of degrees of freedom is estimated via a wavelet decomposition of each of the temporal activation profiles. By inserting the signal-dependent noise term in our modified AIC criterion we insert a mean-variance relationship that deviates from the gaussian noise model. However, this correction term has to be seen only as a rough approximation of the actual effects of the signal dependent noise. Moreover, this term is calculated on a smoothed version of the original data, so that it does not strictly link the estimation error to the average value of the estimation itself.

While the former modification (i.e. the additive noise term) is only an approximation, thus not treated as a deviation from the original white gaussian noise model, the latter aspect of this modified criterion (i.e. the degrees-of-freedom estimation) is crucial for managing the degrees of freedom calculation for highly oversampled signals, such as the sEMG envelope.

All the remaining sEMG processing and synergy extraction techiques have been selected from the most used procedures in the scientific literature [25], [26], regardless of their optimality, in order to increase interpretability of the results with respect to already published studies.

Synergy vectors $W$ have been then ordered by their cosine similarity, and a characteristic set of $W$ vectors for the complete population has been defined coming from each of the identified number of synergies.

### D. Cross-Validation

For testing the possibility of identifying synergies from each of the aforementioned conditions, the $W$ vectors extracted from each of those has been used for reconstructing the data of all the others. The $R^{2}$ values obtained by this reconstruction have been compared with the $95\text{th}$ percentiles of surrogate ones, obtained by reconstructing data with 50 random shuffled versions of the $W$ matrices.

This procedure has been realized in two modalities:

• *trial specific:* In this modality, for each subject, data were cross-validated across trials.
• *$N_{syn}$ specific:* In this modality, all the W vectors extracted from all the subjects and all the trials were used for reconstructing all the data from all subjects and trials. Results from this analysis have then been analysed as a function of the number of synergies related to the W vector used in reconstruction and the number of synergies identified from the reconstructed data by the AIC criterion.

### E. Statistics

The influence of the different factors, namely the *phase* (whole, upward, downward) and the *length* (30 seconds sit-to-stand, 5 times sit-to-stands) has been tested using a three-way ANOVA test with the subject as a random factor. For the ANOVA test, data were organized in a 4-by-$N_{factors} + 1$ matrix, in which the first $N_{factors}$ columns represent the factors, and the last one the parameter under statistical analysis.

Prior to ANOVA testing, data have been transformed using the equation

\begin{equation*}
X^{\prime } = \ln {1 - X} \tag{2}
\end{equation*}

to increase normality in the data and ensure low false positive rate. Post-hoc analysis has been run with a t-test and bonferroni correction. The significance level has been set to $\alpha = 0.05$.

## III. Results

### A. Number of Synergies Extracted

**Fig. 2.**
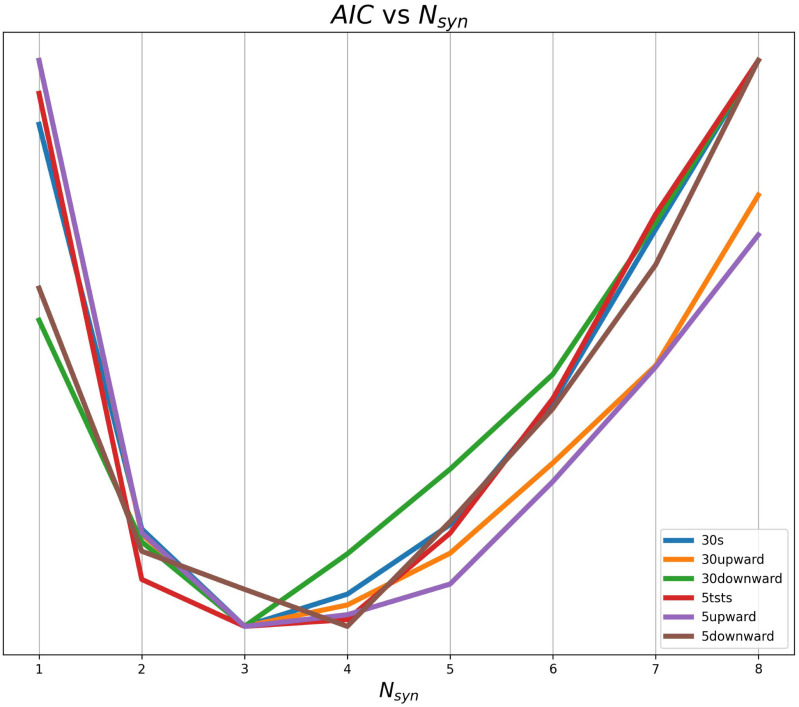
Average $AIC$ vs $N_{syn}$ curves for all the conditions.

The average $AIC$ vs $N_{syn}$ curves are shown in Fig. 2. All the trials, except for *5 downward* show a minimum at $N_{syn}=3$.

**Fig. 3.**
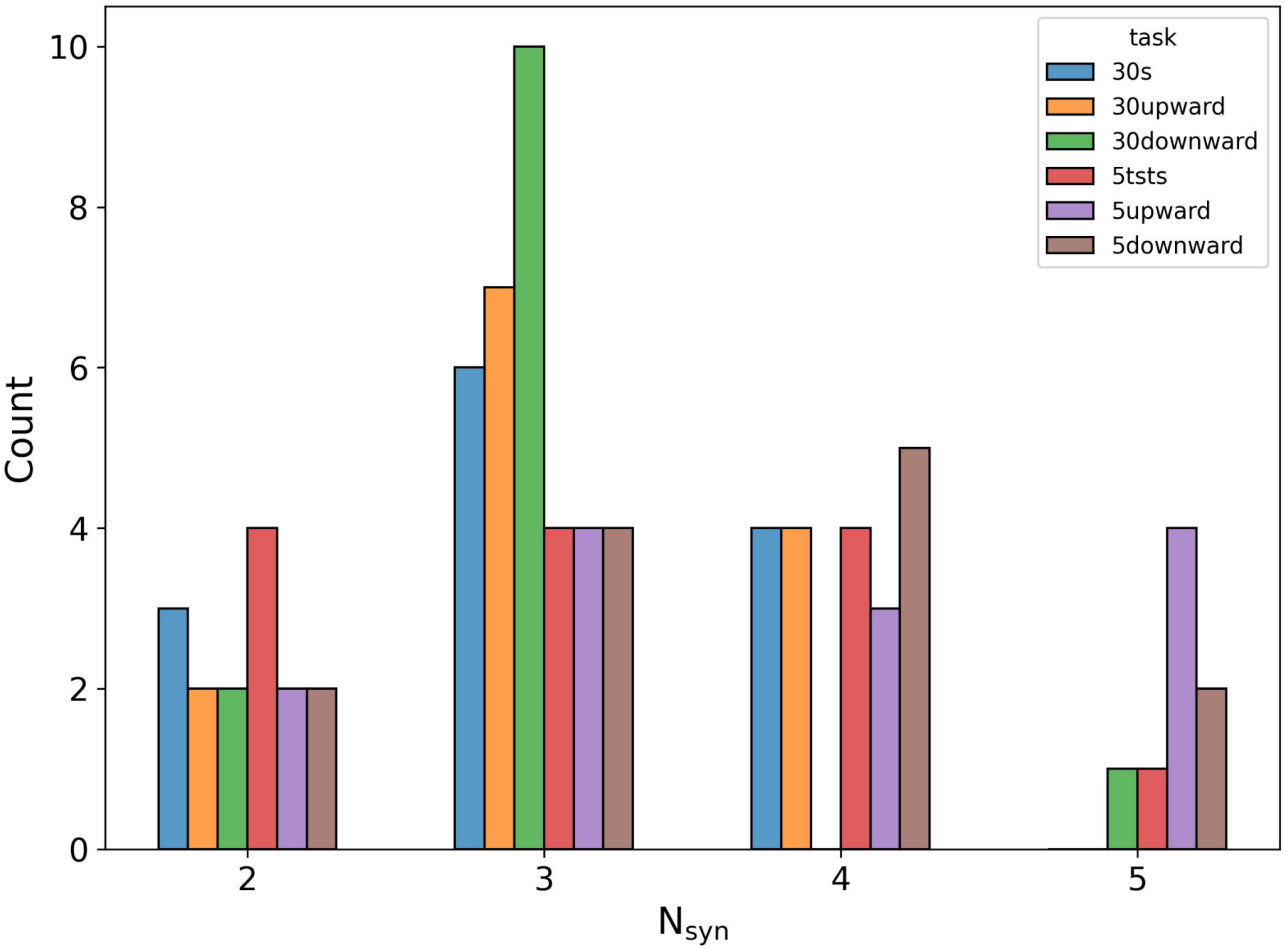
Distribution of the identified $N_{syn}$.

The complete histogram of the identified number of synergies is shown in Fig. 3. Most of the trials were accurately described by 3 synergies, especially for the longer trials. The descriptive statistics of the values is given in Table I.

**TABLE I:** $N_{syn}$ Coming From the Different Trials

**Trial**	**Mean**	**25%**	**Median**	**75%**
*30 s*	3.1	3	3	4
*30upward*	3.2	3	3	4
*30downward*	3.1	3	3	3
*5tsts*	3.2	2	3	4
*5upward*	3.7	3	4	5
*5downward*	3.6	3	4	4

### B. Extracted Synergies

**Fig. 4.**
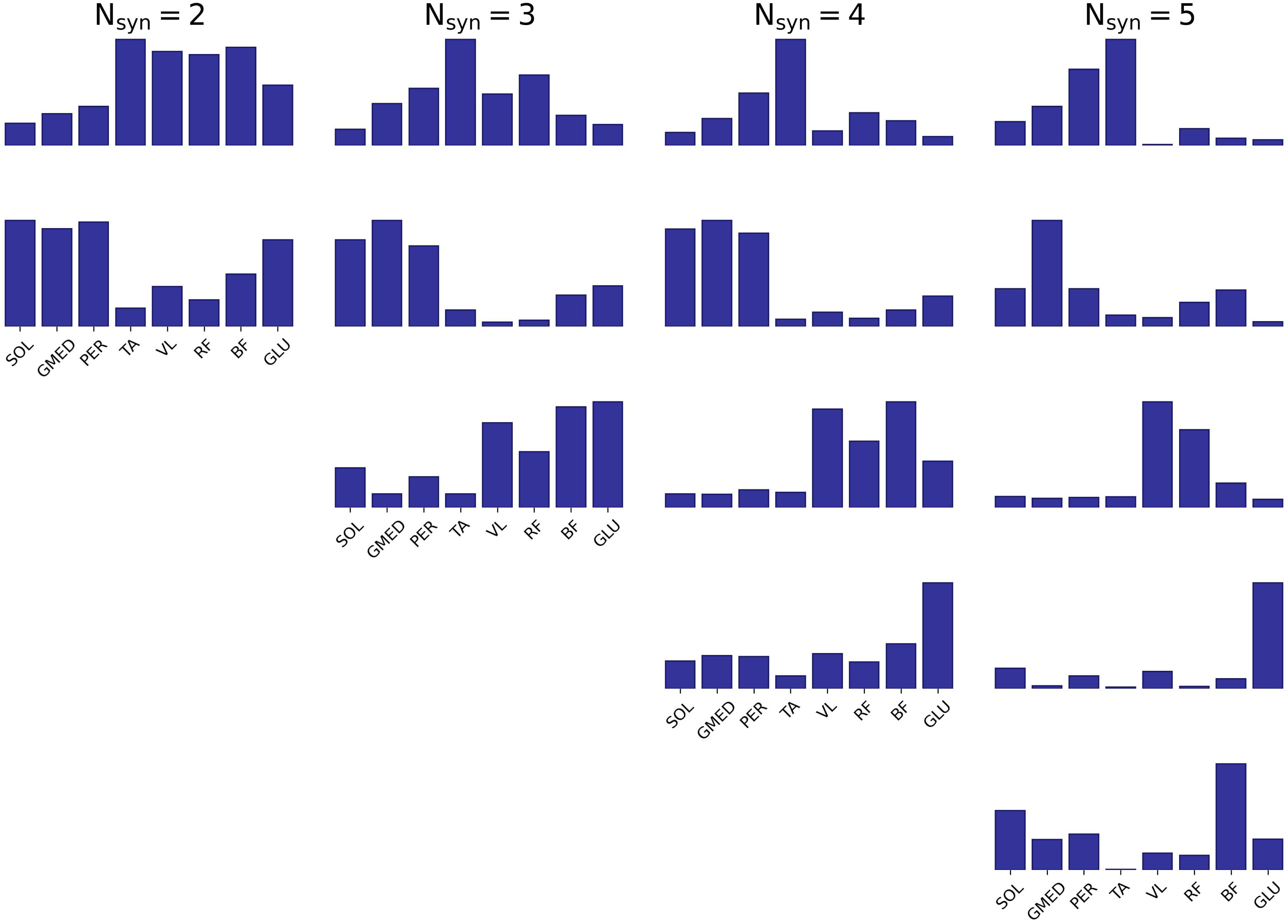
Average (between all the recordings characterized by a specific $N_{syn}$) $W$ vectors for the different $N_{syn}$.

The average synergies coming from the all the identified $N_{syn}$ are shown in Fig. 4.

The spatial structure $W$ shows an increase sparsity when $N_{syn}$ is higher than 3. When quantified as the fraction of samples that are below the mean value, sparsity values are dependent on the number of synergies ($p< 0.05$, ANOVA test), and increasing with the increase of the number of synergies.

In all the sets can however be identified three main functional groups:

• knee extensors with some contributions from knee flexors and hip extensors
• plantar flexors and hip extensors
• muscles acting on the ankle and on the hip

In the trials in which a strong artefact is present, additional modules related to the artefact are found by the optimal $N_{syn}$ selection criterion; these modules are characterized by the strong component of one muscle alone that can be identified for the structures with 4 and 5 synergies.

### C. Cross-Validation Results

**Fig. 5.**
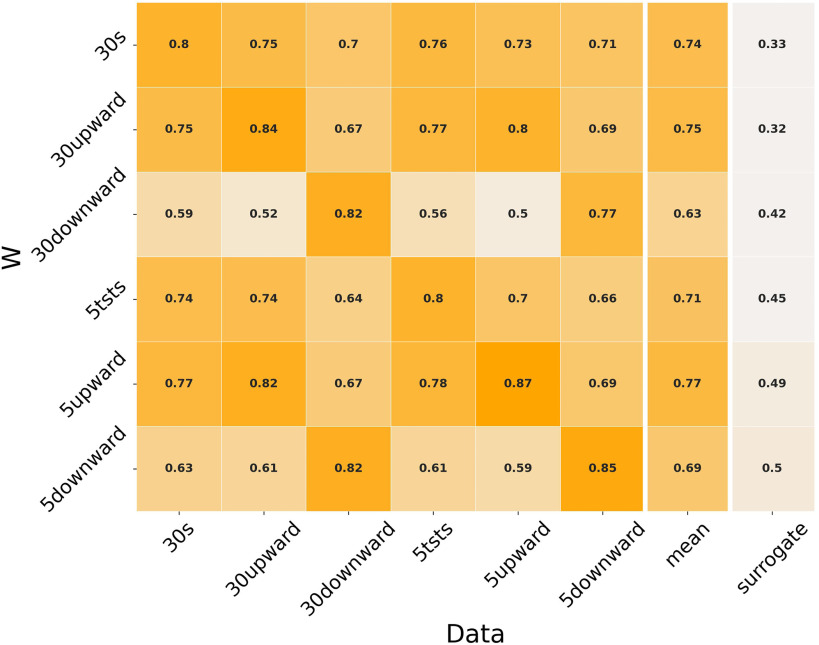
Cross-validation results for the trial-specific analysis.

Cross-validation results coming from the trial-specific analysis are reported in Fig. 5, together with average values and surrogate threshold. The ANOVA analysis shows a significant effect of both *phase* and *length*, as well as of the reconstructed data. Post-hoc analysis shows a significant difference between 30 seconds sit-to-stand and 5 times sit-to-stand based W, with additional significant differences between upward and both the other phases.

**Fig. 6.**
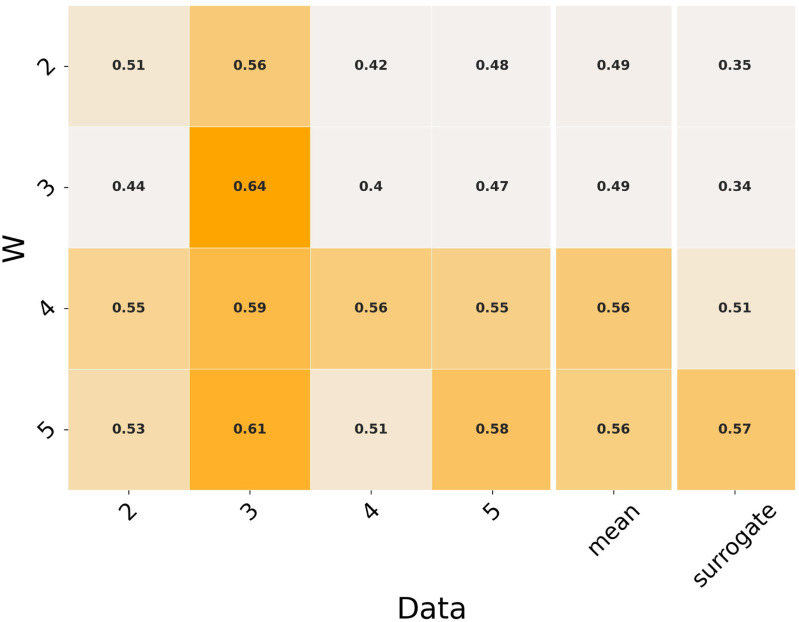
Cross-validation results for the $N_{syn}$-specific analysis.

The same results for the $N_{syn}$-specific analysis are shown in Fig. 6. Statistical analysis shows significant effects of both the number of synergies of the original data and of the reconstruction $W$ set.

## IV. Discussion

The identification of muscle synergies during sit-to-stand clinical test might be a valuable tool for extracting additional information during patient screening, under the hypothesis that it is possible to extract the synergies without affecting the clinical validity of the test by altering its length; results presented here show that it is indeed possible to identify correctly muscle synergies from relatively short recordings during this kind of task. The quality of the identification of muscle synergies has been quantified here by testing their ability to reconstruct the original muscle activation, by considering the $W$ vectors as the basis vectors for the sub-dimensional space in which movement is controlled. This model is widely exploited in the literature and encodes clinically relevant information in the number and composition (typically referred to as *spatial component* of the synergies) of the $W$ vectors themselves. In this framework, if two different sets of $n$ synergy vectors are able to explain correctly muscle activity, then the structure of the vectors is hypothesized to carry equivalent information about motor control. This hypothesis has here been reinforced by the use of a cross-validation procedure aiming to test in a quantitative manner the equivalence of different sets of $W$ vectors, as well as the qualitative comparison given in Fig. 4. When the aim of the analysis is to identify the dimensionality of the motor control structures, most of the tested conditions is able to select the same number of synergies, and this is especially true for the longer trials. This is considered alone to be a clinically relevant information for pathology identification [27], [28], [29] that can be extracted from any of the two clinical tests presented here. It has to be pointed out, however, that this information is unlinked from the quality of the identified synergies, in the sense that even if the dimensionality is maintained between two different trials, the structure of the identified $W$ is not able to yield the same reconstruction performance. This is indeed a result of the two-phase nature of the sit-to-stand-to-sit cycle; a part of the activation might be shared across the *upward* and *downward* phase, while a portion of the activity is different between the two halves of the movement. As a consequence, from the cross-reconstruction $R^{2}$ values, it is reasonable to suppose that the portion of the control strategies that is specific to the *30downward* phase represents a negligible part of the control schemes of the whole movement. Moreover, this phase is highly influenced by the task execution modality; if the subject is asked to perform the movement slowly, it is possible that this instruction generates an additional activity for gravity balancing. As a consequence, being able to extract motor control strategies from the *30upward* phase alone means being able to yield in general a more robust estimation of the motor modules.

The cross-validation analysis for the task-specific reconstruction shows that all the tested couplings are able to yield a reconstruction quality higher than the one obtained by surrogate $W$ vectors, proving that all the identified set of synergy vectors are able to capture at least a significant portion of the control strategies. Moreover, using data coming from the *30 seconds sit-to-stand* test yields similar $R^{2}$ values with respect to the shorter *5 times sit-to-stand*; as a consequence, it can be expected that the same clinically relevant synergy-based information can be extracted from the clinical trials, regardless of their length. In this sense, if the focus of the experimental procedure is the investigation of the motor control strategies, the *30 seconds sit-to-stand* can be considered as a non-optimal test, requiring a longer procedure than actually needed. The results presented here are related to a cohort of healthy people; when dealing with pathological conditions, in which separation between motor modules activity is less evident, longer trials might still be needed for thorough identification of muscle synergies.

From the structures of the $W$ vectors presented in Fig. 4, it is evident how for $N_{syn}$ higher than 3 the additional modules include the activity of one muscle alone. This possibly indicates the presence of artefact on the signal; this artefact is present mostly on the key muscles of the back of the thigh, that typically get in contact with the chair, thus generating a low signal-to-noise ratio. In this analysis, the aim was focused on the identification of synergies without using subjective and advanced filtering technique, simulating the application of such an analysis in a clinical scenario; this means that a portion of the artefact is still present in the signal when undergoing synergy extraction algorithm. The presence of an optimal model selection criterion implies that even a small contribution of the artefact is detected as an additional module. The results presented here, however, show that using longer trials and focusing on the sit-to-stand phase alone ensure the correct identification of the number and structure of the synergies even in the presence of such anomalies in the signal. The presence of the artefact is more relevant when a smaller number of repetitions of the movement is analysed, giving rise to slightly higher $N_{syn}$ values for the *5 times sit-to-stand* trials. Even if artefacts might still be present at low $N_{syn}$, additional modules arise when the effect of artefacts becomes significant on the identification of the synergistic structures; the presence of the optimal criterion, however, allows the identification of this effect from the analysis of the structures themselves, thus helping the neuromechanical assessment of sit-to-stand and increasing the interpretability of the results even in the case of artefact-corrupted, less accurate sEMG recordings; indeed, our aim is the assessment of an optimized method for extracting robust muscle synergies from typical sit-to-stand clinical trials, without any a priori assumption by the experimenter, and even in the presence of movement artefacts on the EMG, that could be present in a typical sit-to-stand trial. This is particularly true for the electrodes placed on the back of the subject (e.g. hamstrings or gluteus), that can easily get in contact with the chair during the test generating a broadband movement artifact that is not removed by a typical EMG band-pass filtering.

The average $N_{syn}$-specific cross-validation shows generally lower $R^{2}$ values for all the tested conditions; even if all these values (except for $N_{syn} = 4$ are higher than the surrogate threshold, they show a non-optimal reconstruction of the original data, even in the self-reconstruction condition (i.e. values on the main diagonal). This can indicate a high degree of inter-subject variability that makes the definition of an inter-subject average set of $W$ vectors an ill-posed problem. However, the structures shown in Fig. 4 are physiologically reasonable and coherent with the biomechanical functions of the muscles under consideration, suggesting that the decrease in the reconstruction quality is mainly due to variability that is outside of the low-dimensional space defined by the muscle synergies. The fact that the sets of synergies characterized by a higher number of components are able to reconstruct all the data characterized by a lower number of original synergies supports the optimality hypothesis on the synergy selection criterion; indeed, this feature of the aforementioned results indicates that additional modules have an additive effect on the synergy groups, thus being identified only when additional components are present (i.e. artefacts), instead of coming from mathematical inaccuracies in the factorization or model selection algorithms.

Results from both subject-specific and average cross-validation analyses show that there is no statistically significant difference between the identification of the synergies using a complete sit-to-stand-to-sit cycle and using the *30upward* phase alone, when the structure of the $W$ vectors is the focus of the analysis. From a neurophysiological standpoint, this implies that the motor modules involved in the sit-to-stand movement are able to control also the opposite movement, determining a common basis to manage the center of mass movement against gravity. This also implies that it is possible to identify muscle synergies only from the *upward* phases, giving rise to the possibility of identifying the sit-to-stand synergies also from different clinical test that include different movements, such as the *timed up and go* tests. Moreover, this last result show that sit-to-stand synergies can also be identified without the need of additional analysis for determining events during the movement, such as movement initiation and termination; in the case of clean sEMG recordings, the use of the complete signal is equivalent to use the sit-to-stand phase alone, reducing the need for additional sensors and additional data processing.

## V. Conclusion

In conclusion, the analysis presented here determined the possibility of identifying muscle synergies from short sit-to-stand instrumented clinical tests. The presence of mathematically optimal methods in the analysis ensures that these results can be applied with validity that is independent of a specific knowledge on sEMG processing techniques. The cross-validation procedure has quantified the differences in terms of the quality of the identified synergy vectors from both a *30 seconds sit-to-stand* and a *5 times sit-to-stand* on a population of healthy subjects. The interpretation of these results highlights the possibility of having an ecological (i.e. short and general purpose) experimental protocol that can be easily applied in the clinical environment.
